# Dynamic Bending Response and Durability of a DSTT-Fabricated MWCNT-Coated Cotton Fabric Sensor

**DOI:** 10.3390/s26185892

**Published:** 2026-09-17

**Authors:** Muhammad Shahbaz, Hiroshi Furuta

**Affiliations:** 1 Graduate School of Engineering, Kochi University of Technology, Kami, Kochi 782-8502, Japan; 256008c@gs.kochi-tech.ac.jp; 2 Research Institute, Kochi University of Technology, Kami, Kochi 782-8502, Japan

**Keywords:** multi-walled carbon nanotubes (MWCNTs), conductive textiles, piezoresistive bending sensor, DSTT, wearable electronics, percolation network, bending durability, hysteresis

## Abstract

Reliable textile sensors must maintain a stable electrical response during repeated bending and across different bending speeds encountered in wearable use. Here, a multi-walled carbon nanotube (MWCNT)-coated cotton fabric fabricated by the drop-casting, sonication, and thermal treatment (DSTT) method was evaluated as a piezoresistive bending sensor against a room-temperature-dried (RT) control at the same coating cycle. The DSTT sensor retained 89.2% of its peak response after 1000 continuous bending cycles, compared with 20.0% for the RT control. Across sequential tests at 1, 2, 4, and 8 mm/s, the DSTT mean peak response varied by 11.1% of the grand mean while maintaining regular waveforms and baseline recovery. Carriage-separation testing gave an average regression sensitivity of approximately 0.58%/mm (R^2^ = 0.982–0.986) and a maximum loading-unloading difference of 8.7% of full scale. A glove-mounted DSTT sensor also produced distinct response levels at manually assigned finger positions of 0°, 45°, and 90°. For the selected specimens, these results demonstrate greater dynamic response stability for DSTT than for RT under the tested protocols, together with discrimination among manually assigned finger positions in the glove demonstration. The speeds were applied in a fixed order; therefore, the effects of bending speed and cumulative cycle history cannot be separated.

## 1. Introduction

Textile-based strain sensors combine the sensing function of flexible electronics with the mechanical flexibility, breathability, and comfort of ordinary fabric, and they have drawn wide interest for human motion detection and physiological monitoring [1]. Matching sensor output to the speed and complexity of real human movement remains a key design problem [2], and reviews of carbon-based and textile strain sensors have summarized the materials, sensing mechanisms, and fabrication routes that distinguish this sensor class, including surveys of electronic-textile coating methods [3,4,5,6], demonstrations such as carbon nanotube (CNT)-coated cotton for motion detection, knitted sensor arrays, and screen-printed graphene/multi-walled carbon nanotube (MWCNT) gloves for gesture recognition [7,8,9], and comparisons of cotton and CNTs against metallic or polymer alternatives on the basis of stability, weight, and corrosion resistance [10,11,12,13,14]. Several coating routes have been reported for CNT-coated cotton specifically, including drop-casting, dip-coating, dyeing, functionalization-assisted deposition, layer-by-layer assembly, and inkjet printing, and a number of these also demonstrate strain- or motion-sensing performance on the coated fabric [15,16,17,18,19,20,21,22,23,24].

Beyond fabrication and static conductivity, the dynamic behavior of these sensors is the focus of the present paper. The conduction mechanism is governed by the internal conductive network and by tunneling resistance between neighboring CNTs, both of which respond to the instantaneous spacing between nanotubes [25,26,27]. Cyclic durability over hundreds to a few thousand bending cycles is a standard test, with reported examples ranging from 1000 to 4000 cycles and including angle-resolved distinction between bending states in several designs [28,29,30,31,32,33]. Hysteresis is usually attributed to viscoelastic energy loss during bending and recovery, and has been reduced or deliberately exploited in different sensor designs [34,35,36], while rate dependence has been demonstrated directly in textile-based inductive sensors tracking fast joint movement [37]. Few studies have quantitatively evaluated cyclic durability, response across different bending speeds, and bending-deformation hysteresis together for CNT-coated cotton fabrics in bending mode; partial coverage exists elsewhere, for example, a printed graphene/CNT/thermoplastic polyurethane (TPU)-fabric strain sensor combining 3000-cycle durability with an explicit frequency sweep [38]. This makes it difficult to know whether a reported output maps reliably onto the deformation that produced it.

In our previous work, we introduced the drop-casting, sonication, and thermal treatment (DSTT) method, combining drop-casting, sonication during deposition, and mild thermal treatment into a single process for fabricating MWCNT-coated conductive cotton fabrics [14]. Compared to three conventional routes (room-temperature drying, vacuum drying, and sonication alone), DSTT produced the lowest sheet resistance, reaching 0.072 ± 0.004 kΩ/sq. at ~30 wt.% MWCNT loading, and this performance was reproducible across fabric sizes and stable over 16 weeks of ambient storage with periodic manual bending [14]. That study characterized fabrication and static electrical performance only; it did not examine how the DSTT fabric behaves as an active sensor under dynamic loading. The present paper provides that characterization as a direct continuation of the fabrication work. The DSTT Coating 6 specimen from the earlier study (six coating cycles, ~30 wt.% MWCNT loading), which exhibited the lowest sheet resistance among the fabrication routes examined [14], is tested here as a bending sensor on a custom linear-actuator rig, alongside a room-temperature-dried (RT) control at the same coating cycle (RT Coating 6), to determine whether the morphological advantage of DSTT found for static sheet resistance [14] extends to dynamic sensing. The tests quantify the cyclic response at four bending speeds, durability across 1000 continuous bending cycles, single-cycle waveform comparison across the tested speeds, the response to bending deformation and its hysteresis, and a glove-mounted demonstration distinguishing finger-bending angles of 0°, 45°, and 90°. The contribution of the present study is the combined characterization of cyclic response retention, response across a sequential bending-speed protocol, and bending–bending-back hysteresis for selected DSTT-coated cotton sensors, with an RT specimen at the same coating cycle providing a direct dynamic-performance comparison.

## 2. Materials and Methods

### 2.1. Sample Fabrication

The conductive fabrics were fabricated by the DSTT method described in detail in our previous work [14]: cotton fabric (moonfarm, Southern Wind Co., Ltd., Saku, Japan) was drop-cast with a commercial multi-walled carbon nanotube (MWCNT) dispersion in ethanol (5 wt.%, product MW-I, Meijo Nanocarbon Co., Ltd., Nagoya, Japan; MWCNT diameter 10–40 nm), with 500 ± 10 μL applied per coating cycle, while under indirect sonication using an ultrasonic bath (SND Co., Ltd., Suwa, Japan; 38 kHz, 30 min per cycle, bath temperature displayed by the sonication apparatus at approximately 50 °C), then vacuum-heated at 100 °C for 1 h (BV-001 bell-jar vacuum oven, SIBATA Scientific Technology Ltd., Soka, Japan, operated at maximum vacuum, −0.1 MPa gauge) to consolidate the coating. Sonication during deposition assists the de-bundling of CNTs [39], which in this fabric is expected to aid their penetration into the porous fiber structure, although extended sonication is also known to shorten CNTs and degrade the mechanical and thermal properties of the resulting composite, which is why the process was limited to 30 min per cycle here [40], and the thermal step drives the composite further above the percolation threshold with each successive coating cycle [14]. Six coating cycles were applied, giving the DSTT Coating 6 sample used here, at an MWCNT loading of ~30 wt.%, the condition that produced the lowest sheet resistance among all fabrication routes examined in the fabrication study [14]. The control sample (RT Coating 6) was prepared the same way but dried under ambient laboratory conditions for more than 24 h, without sonication or thermal treatment, at room temperature with ambient relative humidity ranging from approximately 50% to 70% [14].

### 2.2. Sensor Construction

**Figure 1:**
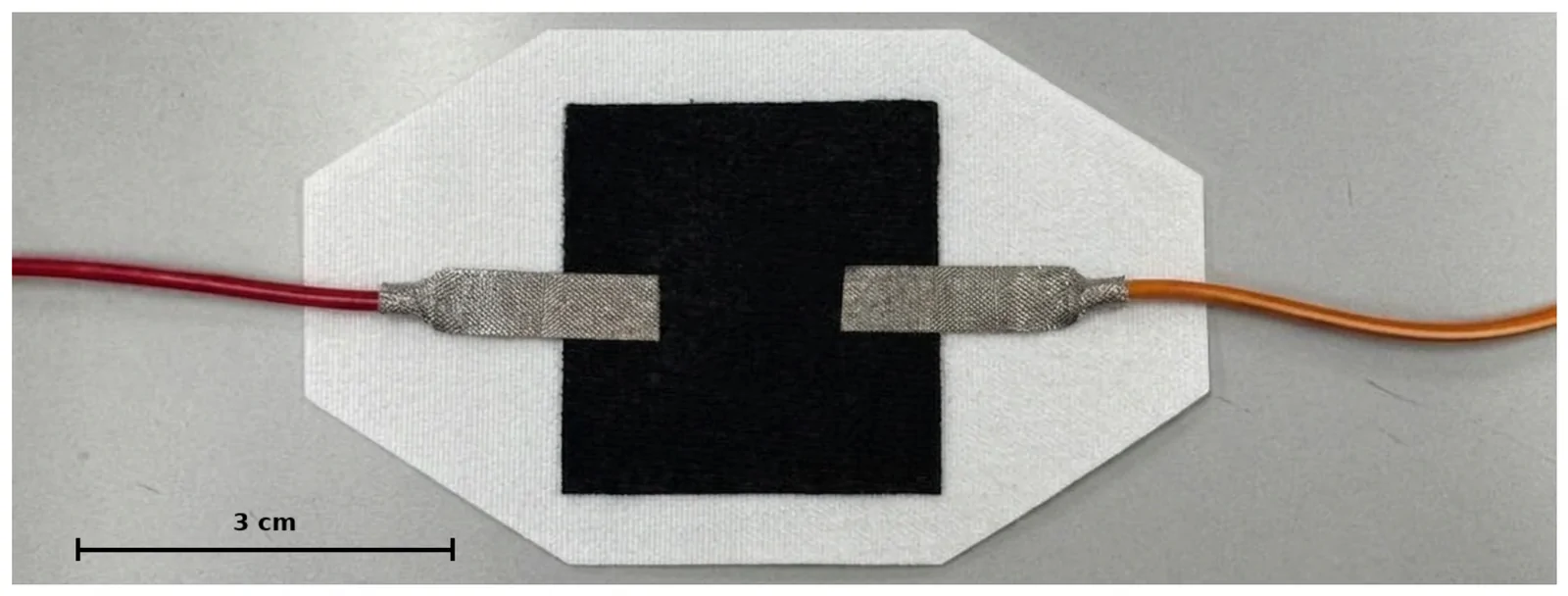
Photograph of sensor construction, showing the DSTT-coated cotton fabric mounted on gauze tape with conductive fabric tape electrodes at opposite edges and lead wires for resistance measurement. DSTT: drop-casting, sonication, and thermal treatment.

Two sensor configurations were used, matched to the two test geometries described in Section 2.3. For the multi-speed and cyclic-durability tests, DSTT- and RT-coated cotton samples measuring 2.5 × 2.5 cm^2^ were used; the DSTT specimen was additionally used for the carriage-separation test. Each end of the fabric was affixed directly to a rig carriage with commercially available double-sided tape, and two electrode contacts were applied at opposite edges, approximately 2.5 cm apart, using conductive fabric tape (uxcell, Hong Kong, China) connected to lead wires for resistance measurement. For the glove-mounted finger-bending test, a separate DSTT-coated sample measuring 3.0 × 3.0 cm^2^ (Figure 1) was mounted on commercially available gauze tape, which served as a support for handling and attachment to the finger; two electrode contacts were applied at opposite edges, 2 cm apart, using the same conductive fabric tape and lead-wire arrangement. Because the glove configuration used a different electrode spacing and deformation geometry, its response is presented as a wearable angle-discrimination demonstration rather than a direct quantitative comparison with the carriage-separation sensitivity.

### 2.3. Bending Test Rig and Data Acquisition

Controlled bending tests were performed on a custom-built rig based on a stepper-motor-driven linear rail actuator (Akozon, Shenzhen, China). The stepper motor was controlled by an Arduino UNO-compatible microcontroller board (ELEGOO, Shenzhen, China) through a DRV8825 stepper motor driver module (Youmile, Shenzhen, China), allowing the actuator speed and travel to be programmed. The sensor was affixed at both ends with double-sided tape, with one end fixed and the other attached to the moving carriage of the actuator. Reducing the separation between the two carriages caused the fabric to arch; this carriage separation, L, is defined as the distance between the fixed and moving carriages, with L_0_ denoting the carriage separation at the flat, unbent starting configuration; L decreases as the fabric is bent more tightly, so the deformation is given by L_0_ − L. The sensor resistance was read out through a voltage divider with a fixed reference resistor using the Arduino UNO’s onboard 10-bit analog-to-digital converter (ADC) (0–5 V input range), and the time-stamped measurements were logged over a universal serial bus (USB) serial connection to a computer for subsequent analysis. In the multi-speed dataset, consecutive timestamps were separated by 0.050 s, corresponding to a sampling rate of 20 Hz. Initial resistance (R_0_), measured with a two-probe digital multimeter (TST-KJ830, OHM Electric Inc., Yoshikawa, Japan), was approximately 100 Ω for both DSTT sensors (2.5 × 2.5 cm^2^ and 3.0 × 3.0 cm^2^) and approximately 5.6 kΩ for the RT sensor (2.5 × 2.5 cm^2^); the fixed reference resistor in each voltage divider was matched to the corresponding sensor’s R_0_ (100 Ω for DSTT, 5.6 kΩ for RT) to maximize divider sensitivity.

With R_ref_ matched to R_0_, the 10-bit ADC corresponds to nominal quantization increments in ΔR/R_0_ of approximately 0.39 percentage points near baseline and 0.44 percentage points near the DSTT peak response. These increments are substantially smaller than the principal response amplitudes and response differences evaluated in this study; changes approaching the quantization level are interpreted conservatively.

Sensor resistance was calculated from the measured divider output voltage as

Rsensor = R_ref_ × Vout/(Vin − Vout), (1)

where Vin = 5 V and R_ref_ is the fixed reference resistor; Equation (1) corresponds to Vout measured across the sensor leg of the voltage divider. Because the DSTT sensor’s resistance (≈100 Ω) is low and was measured with a two-probe configuration, lead and contact resistance may contribute to the measured R_0_. Accordingly, ΔR/R_0_ is interpreted consistently as the relative change in the measured sensor resistance, including contributions from lead and contact resistance.

The stepper motor drove a mini linear rail guide slide actuator stepper motion stage (50 mm rated stroke, with a 2 mm reserved stroke-protection margin at each end) through the DRV8825 driver. The actuator travel used in testing was approximately 21 mm per half-cycle, based on manual measurement of the carriage displacement, which was approximately 21–22 mm, corresponding to approximately 42 mm per complete bending-release cycle and remaining well within the stage’s rated travel.

**Figure 2:**
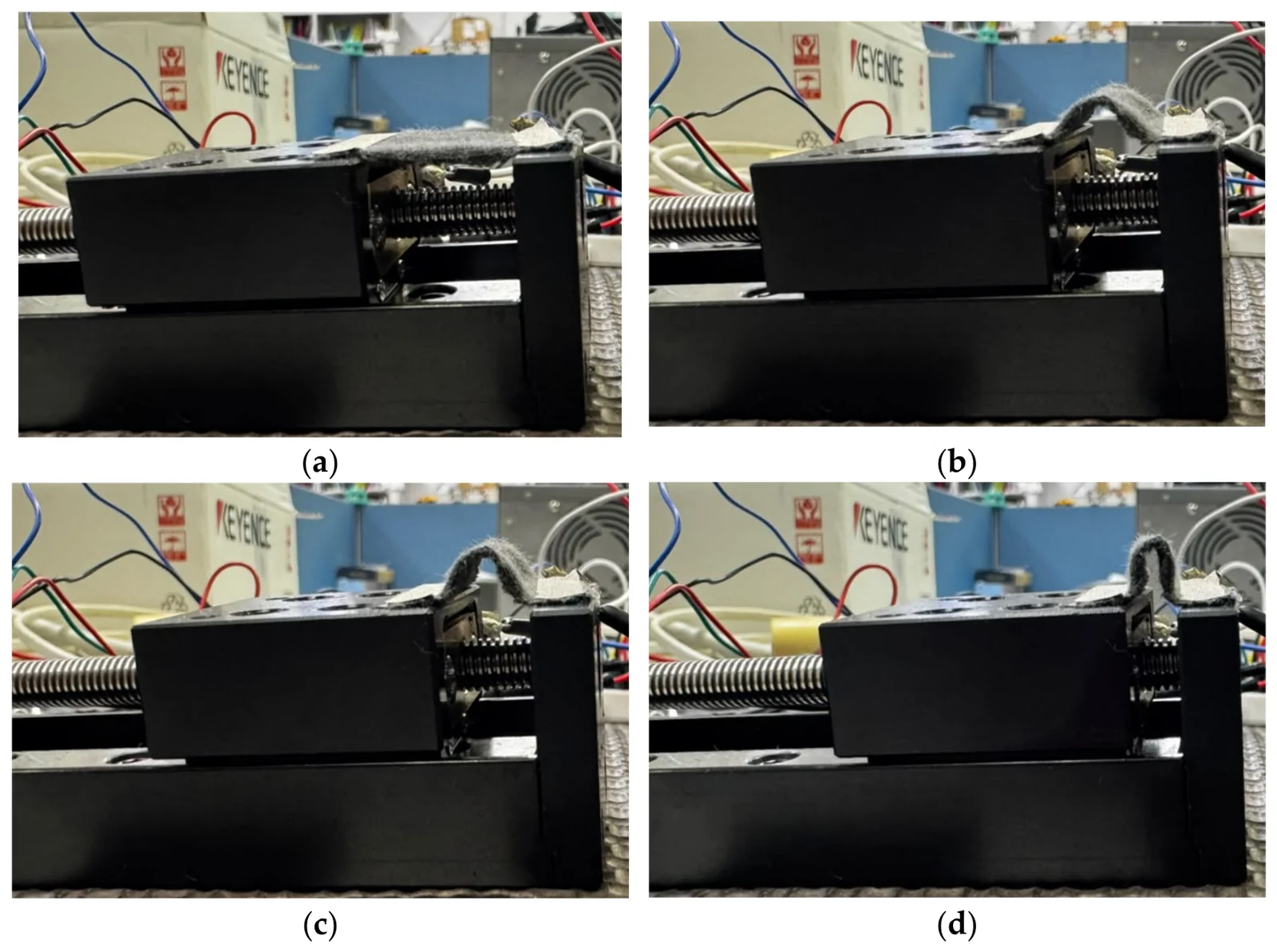
Representative photographs of the rig-mounted sensor at carriage separations of (**a**) 25 mm (flat configuration), (**b**) 18 mm, (**c**) 11 mm, and (**d**) 4 mm.

Figure 2 shows representative sensor configurations at carriage separations of 25, 18, 11, and 4 mm, illustrating progressive bending as the carriage separation decreases. Carriage separation, L, is the directly controlled geometric variable; these photographs illustrate the corresponding configurations without establishing a quantitative conversion to bending angle or curvature.

All results are reported as the relative resistance change ΔR/R_0_, where R_0_ is the resistance of the flat, unbent sensor at the beginning of each test. All tests were performed at room temperature and 55–60% relative humidity.

### 2.4. Test Protocols

Four experimental protocols were used. For the two rig-mounted specimens, the multi-speed dynamic response was performed first, with programmed actuator speeds of 1, 2, 4, and 8 mm/s applied as consecutive segments in ascending order, followed by the cyclic-durability test after a rest interval of more than 24 h. Because the speed segments were applied consecutively on the same specimen, speed and cumulative cycle history are not independently separable in this protocol. The carriage-separation dependence and hysteresis test were then performed on the DSTT specimen only. Separately, a glove-mounted DSTT specimen was used for the angle-stepped bending demonstration. Single-cycle waveforms were extracted from the multi-speed dataset for waveform comparison.

Multi-speed dynamic response. Both the DSTT and the RT sensors were subjected to consecutive segments of repeated bending-release cycles at programmed actuator speeds of 1, 2, 4, and 8 mm/s, over a carriage-separation range of L_0_ ≈ 25 mm to L ≈ 4.0 mm at each speed, recorded as a continuous time series. Identical protocols were applied to both samples.

Cyclic durability. Both sensors were subjected to 1000 continuous bending-release cycles at a fixed actuator speed of 4 mm/s, over a carriage-separation range of L_0_ ≈ 25 mm to L ≈ 4.0 mm, with no dwell period at the extremes of carriage travel, and ΔR/R_0_ recorded for every cycle.

Single-cycle waveform analysis. Individual bending-release cycles of the DSTT sensor were extracted at each of the four actuator speeds for a direct comparison of the waveform shape, duration, and peak response.

Carriage-separation dependence and hysteresis. The DSTT sensor was driven at an actuator speed of 4 mm/s through one complete bending and bending-back sweep, from L_0_ ≈ 25 mm to L ≈ 4.0 mm and back. ΔR/R_0_ was continuously recorded in both directions.

Glove-mounted bending-angle test. The DSTT sensor was mounted on the finger of a commercially available glove and connected to the same Arduino-based data acquisition setup. The finger was manually stepped through 0°, 45°, and 90° bending angles; verified against a printed angle template; and held at each angle for approximately 5 s before moving on to the next. The 0° → 45° → 90° → 45° sequence was repeated five times within a single continuous 128 s recording, and reference photographs of the sensor position at each angle were taken during the test. Because the repeated angle presentations occurred within one continuous measurement rather than as independent trials, they are treated as repeated observations within a single experiment rather than independent replicates. The stated angles therefore represent manually assigned approximate positions rather than instrumented angular measurements. This test serves simultaneously as a bending-angle characterization and as the finger-bending demonstration.

### 2.5. Data Processing and Analysis

Peak values for each bending cycle were identified from the recorded ΔR/R_0_ data using SciPy’s peak-detection algorithm (scipy.signal.find_peaks). Minimum peak distances of 840, 420, 210, and 105 samples were used for the 1, 2, 4, and 8 mm/s segments, respectively, based on the expected cycle spacing and to prevent multiple peak detections within a single bending cycle. For each identified cycle, the peak response was taken as the maximum ΔR/R_0_ value within the corresponding cycle window, while the baseline was taken as the minimum value within that cycle. For the 1000-cycle durability test, the first and final 20 cycles were defined as cycles 1–20 and 981–1000, respectively. All reported ± values are sample standard deviations (SD).

Retention was calculated as

Sret = Sfinal/Sinitial × 100%,(2)

where Sfinal and Sinitial are the mean peak ΔR/R_0_ over the final and initial 20 cycles, respectively.

The coefficient of variation (CV) was calculated as

CV = σS/Smean × 100%,(3)

where σS and Smean are the standard deviation and mean of the peak response across all 1000 cycles.

Peak variation across speeds was calculated as

ΔSspeed = (Smax − Smin)/Sgrand,mean × 100%,(4)

where Smax and Smin are the highest and lowest mean peak responses among the four tested speeds, and Sgrand,mean is their grand mean, computed as the unweighted arithmetic average of the four per-speed means. Because the speeds were applied sequentially, this grand mean is used only as a descriptive reference for the reported speed-segment variation.

For the carriage-separation test, bending and bending-back values were paired by carriage separation, L, recorded at each measurement point during the continuous sweep. Maximum hysteresis was calculated as

Hmax = max|Sbend(L) − Sreturn(L)|/SFS × 100%,(5)

where Sbend(L) and Sreturn(L) are the ΔR/R_0_ values at matched L during bending and bending-back, respectively. Full-scale response was defined as

SFS = Smax,sweep − Smin,sweep,(6)

where Smax,sweep and Smin,sweep are the maximum and minimum ΔR/R_0_ values recorded across the full bending and bending-back sweep.

Linear regression of ΔR/R_0_ against L was performed using SciPy’s linregress function. All statistical analysis was conducted in Python 3.12.13 (pandas 2.2.2, NumPy 2.0.2, SciPy 1.16.3). Figures were generated in Google Colab (Python 3.12.13) using matplotlib 3.10.0, with pandas and NumPy for data handling, SciPy’s linregress for the regression lines, and Pillow 11.3.0 for image handling.

Reproducibility of the static electrical properties of DSTT-coated cotton fabrics across coating cycles and fabric sizes was established previously using multiple specimens [14]. The present work was designed as a detailed dynamic characterization of selected Coating 6 sensors: one DSTT specimen and one RT specimen (2.5 × 2.5 cm^2^) were used for the multi-speed and cyclic-durability tests; the DSTT specimen was additionally used for the carriage-separation and hysteresis test; and a separate DSTT specimen (3.0 × 3.0 cm^2^) was used for the glove demonstration. The repeated cycles therefore quantify within-specimen dynamic repeatability; the preceding study supports reproducibility of static electrical properties [14], rather than between-specimen reproducibility of dynamic sensor performance.

## 3. Results

### 3.1. Multi-Speed Dynamic Response: DSTT Versus RT

**Figure 3:**
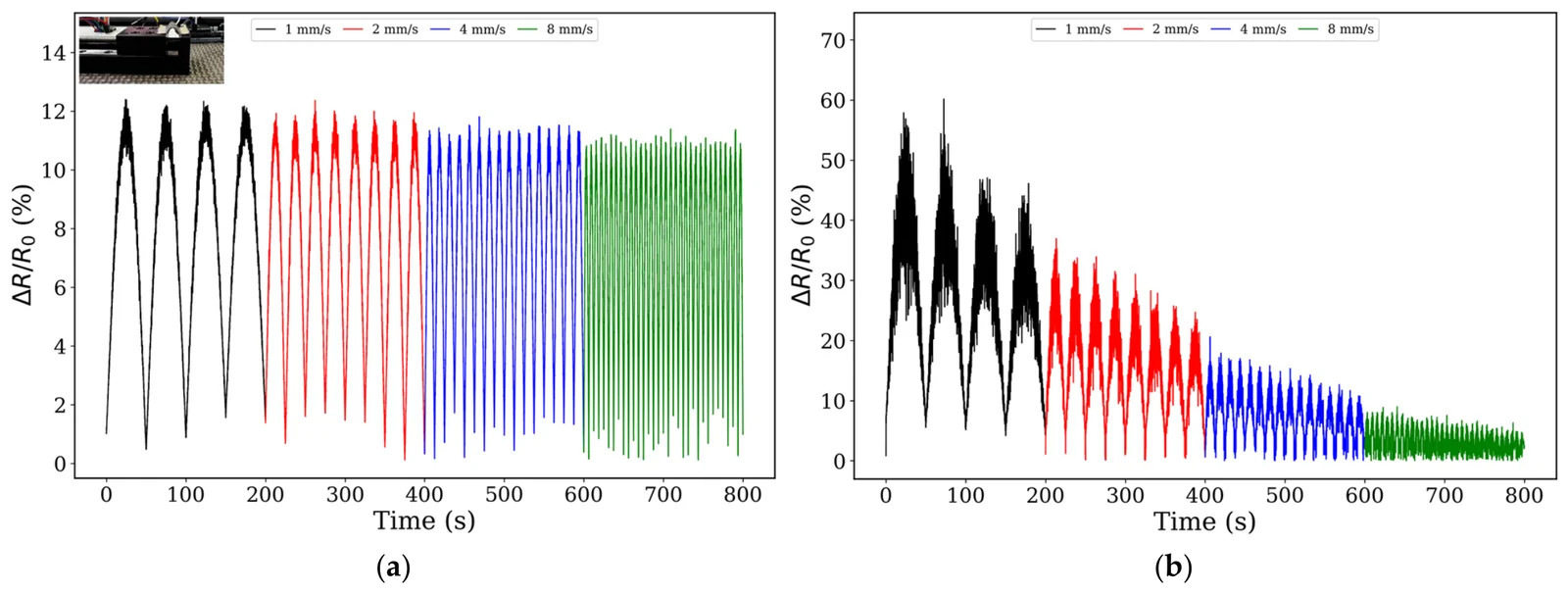
Dynamic bending response in consecutive segments at 1, 2, 4, and 8 mm/s for (**a**) the DSTT Coating 6 sensor (inset: the linear-actuator bending rig) and (**b**) the RT Coating 6 control, plotted as ΔR/R_0_ versus time. RT: room-temperature-dried control.

Figure 3 compares the dynamic response of the DSTT and RT sensors over consecutive bending cycles at 1, 2, 4, and 8 mm/s. The DSTT sensor produces clean, regular waveforms with a stable baseline that is recovered after each cycle. Mean peak responses were 12.27 ± 0.23%, 11.95 ± 0.20%, 11.40 ± 0.15%, and 10.98 ± 0.16% at 1, 2, 4, and 8 mm/s, respectively (Table 1). The difference between the highest and lowest mean peaks was 11.1% of the unweighted grand mean. Thus, despite the eight-fold change in actuator speed, the selected DSTT specimen maintained a comparatively narrow response range and regular waveform morphology throughout the sequence. The 11.1% variation characterizes the response across the sequential speed protocol and includes any contribution from cumulative cycle history; it should not be interpreted as an independently measured bending-speed effect.

The RT sensor behaves differently under the identical protocol. Its response at the lowest speed is several times higher than that of the DSTT sensor, but the peak amplitude decays visibly from cycle to cycle and the waveform is substantially noisier. The mean peak response decreased from 52.81 ± 7.26% at 1 mm/s to 30.67 ± 4.29% at 2 mm/s, 14.96 ± 2.56% at 4 mm/s, and 6.72 ± 1.13% at 8 mm/s (n = 4, 8, 16, and 32 cycles). The range between the highest and lowest RT mean peak responses corresponded to 175% of the grand mean. Each speed segment lasted 200 s, and 28 complete bending cycles had been performed before the 8 mm/s segment began. Because the speeds were applied in a fixed order, the RT decline cannot be assigned uniquely to speed and is interpreted together with cumulative cycle-history effects. The key contrast is therefore not raw amplitude but dynamic stability: DSTT maintains a regular waveform and recovered baseline, whereas the RT response progressively loses amplitude during the same sequential protocol.

### 3.2. Cyclic Durability Across 1000 Bending Cycles

**Figure 4:**
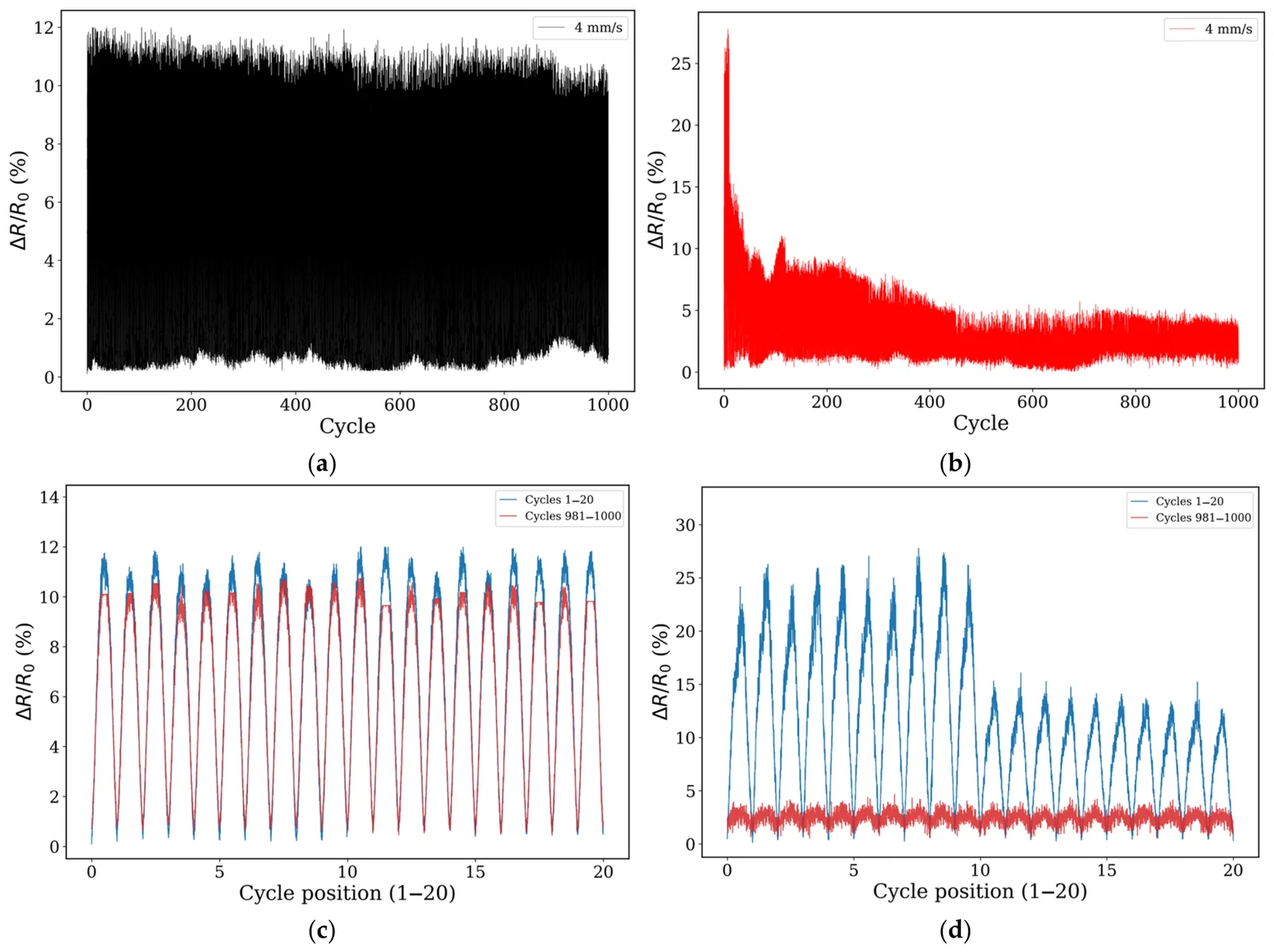
ΔR/R_0_ across 1000 continuous bending cycles at 4 mm/s for (**a**) the DSTT Coating 6 sensor and (**b**) the RT Coating 6 control; (**c**,**d**) the first and last 20 cycles of the corresponding sensors overlaid. Peak-response retention, calculated from the final and initial 20-cycle mean peaks, was 89.2% for DSTT and 20.0% for RT.

Figure 4 shows the response of both sensors across 1000 continuous bending cycles at a fixed speed of 4 mm/s. The DSTT sensor retained 89.2% of its peak response across 1000 cycles (mean peak of the final 20 cycles relative to the initial 20 cycles), with a peak coefficient of variation of 4.3% across the full test. The apparent baseline shift between the first and last 20 cycles was +0.22 percentage points, below the nominal ADC quantization increment near baseline; this small change is therefore interpreted conservatively. Figure 4c overlays the first and last 20 cycles directly, showing the close agreement of the two amplitude bands.

The RT control shows substantially greater degradation. After a rest interval of more than 24 h following the multi-speed sequence, its initial 20-cycle mean peak in the durability test was 20.24 ± 6.04%, higher than the 14.96% mean peak recorded at the same nominal speed during the earlier sequence. This difference is consistent with partial recovery of the RT response between tests. Thereafter, the amplitude declined markedly, ending at 20.0% of its initial peak response, with a peak coefficient of variation of 50.1% across the full 1000-cycle test, more than ten times that of the DSTT sensor. Figure 4d makes this contrast explicit by overlaying the first and last 20 cycles. At the end of the test, the RT sensor retained 20.0% of its initial peak response, whereas the DSTT sensor retained 89.2%. This result extends the long-term storage and manual-bending stability established for DSTT coatings in the fabrication study [14] to sustained machine-controlled cyclic deformation. Table 2 summarizes the dynamic-performance metrics.

Before- and after-test unbent resistances were measured using a two-probe digital multimeter. The post-test measurement was taken after completion of the cyclic test, with the fabric placed on a flat surface and the electrode contacts remaining attached. R_0_ denotes the initial unbent resistance used for response normalization; the post-test value is reported separately as the final unbent resistance. The apparent baseline shift was calculated from the difference between the mean cycle-minimum ΔR/R_0_ values over the final and initial 20 cycles recorded on the bending rig. These measurements were obtained under different mechanical and measurement conditions and are therefore reported separately. CV: coefficient of variation; ADC: analog-to-digital converter.

### 3.3. Single-Cycle Response Across Sequential Speed Segments

**Figure 5:**
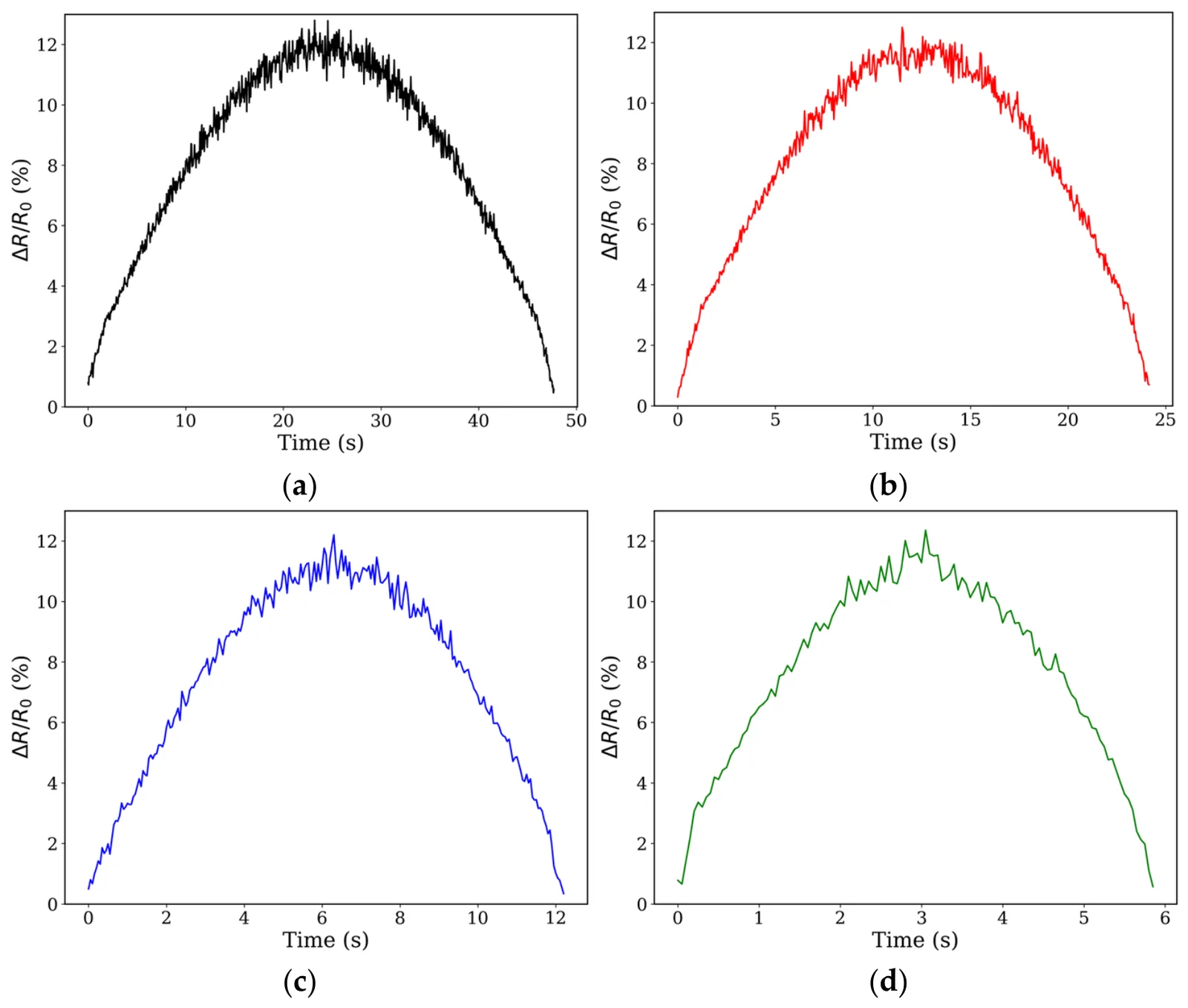
Single-cycle ΔR/R_0_ waveforms of the DSTT Coating 6 sensor at 1, 2, 4, and 8 mm/s, showing similar arc-shaped profiles and cycle durations that scale inversely with actuator speed. The highest and lowest mean peak responses differ by 11.1% of their grand mean. (**a**) 1 mm/s; (**b**) 2 mm/s; (**c**) 4 mm/s; (**d**) 8 mm/s.

To examine the DSTT response at the four actuator speeds more closely, single bending-release cycles were extracted from the multi-speed recording (Figure 5). At every speed, the waveform retains the same smooth, arc-shaped rise-and-fall profile, while cycle duration decreases as expected with increasing actuator speed. The highest and lowest mean peak responses differ by 11.1% of their grand mean (Section 3.1).

The similar waveform profiles across the four tested speeds show that the DSTT response remained dynamically regular throughout the sequence. Because the speeds were applied in fixed ascending order, the modest amplitude difference is reported descriptively rather than interpreted as an isolated rate coefficient.

### 3.4. Carriage-Separation Dependence and Hysteresis

**Figure 6:**
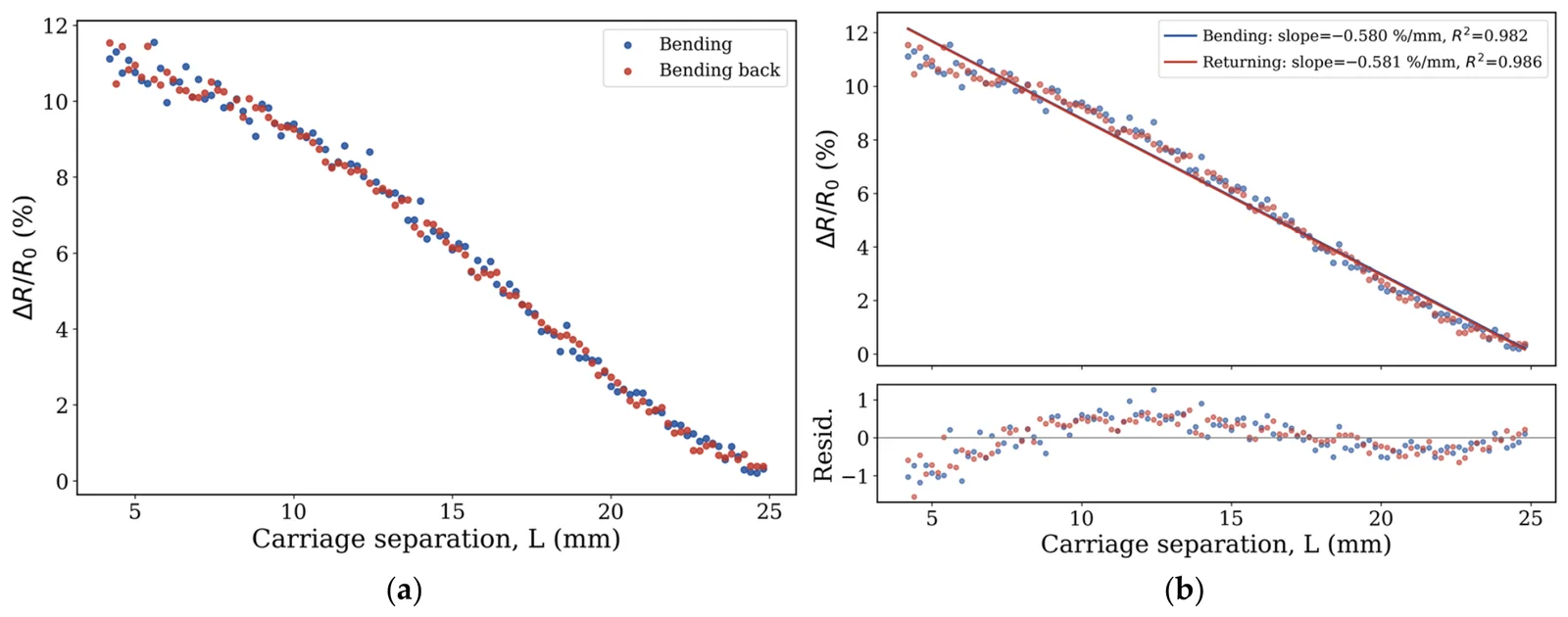
ΔR/R_0_ of the DSTT Coating 6 sensor as a function of carriage separation during bending and bending-back. (**a**) All measured points, showing the full hysteresis dataset; (**b**) the same full dataset with linear regression lines, sensitivity, R^2^, and residuals shown for each direction. The reported maximum hysteresis of 8.7% of full scale was obtained from this single complete sweep at 4 mm/s.

Figure 6 shows ΔR/R_0_ of the DSTT sensor as a function of carriage separation, L, during bending and bending-back over the full tested range (L ≈ 4.0–25 mm). The response increased monotonically as the carriage separation decreased and the bend became tighter. Linear regression gave slopes of −0.580%/mm (R^2^ = 0.982) during bending and −0.581%/mm (R^2^ = 0.986) during bending-back, corresponding to an average rig-specific sensitivity of approximately 0.58% per mm of carriage-separation change (L_0_ − L). The regression slopes slightly exceed the end-to-end slopes because of the modest nonlinearity over the full sweep. The close bending and bending-back slopes demonstrate that carriage separation provides a consistent calibration variable for this test geometry. Because local strain or curvature was not measured directly, the result is reported in the experimentally controlled %/mm unit rather than converted to an assumed gauge factor. Accordingly, Figure 6 represents a resistance-based calibration against controlled carriage separation; it does not establish a conductivity–strain relationship. The reported sensitivity characterizes this carriage-separation configuration and should not be directly compared numerically with strain-based gauge factors or angle- and curvature-based sensitivities without an independently established geometric relationship.

The bending and bending-back data nearly overlap throughout the range. The maximum difference was 0.98 percentage points, corresponding to 8.7% of the full-scale response, while the mean difference was 0.25 percentage points. The closely matched regression slopes and limited separation between the bending and bending-back responses indicate limited directional dependence within the tested range. The reported maximum hysteresis of 8.7% of full scale was obtained from this single complete sweep at 4 mm/s.

### 3.5. Angle-Stepped Bending Response: Finger-Bending Demonstration

**Figure 7:**
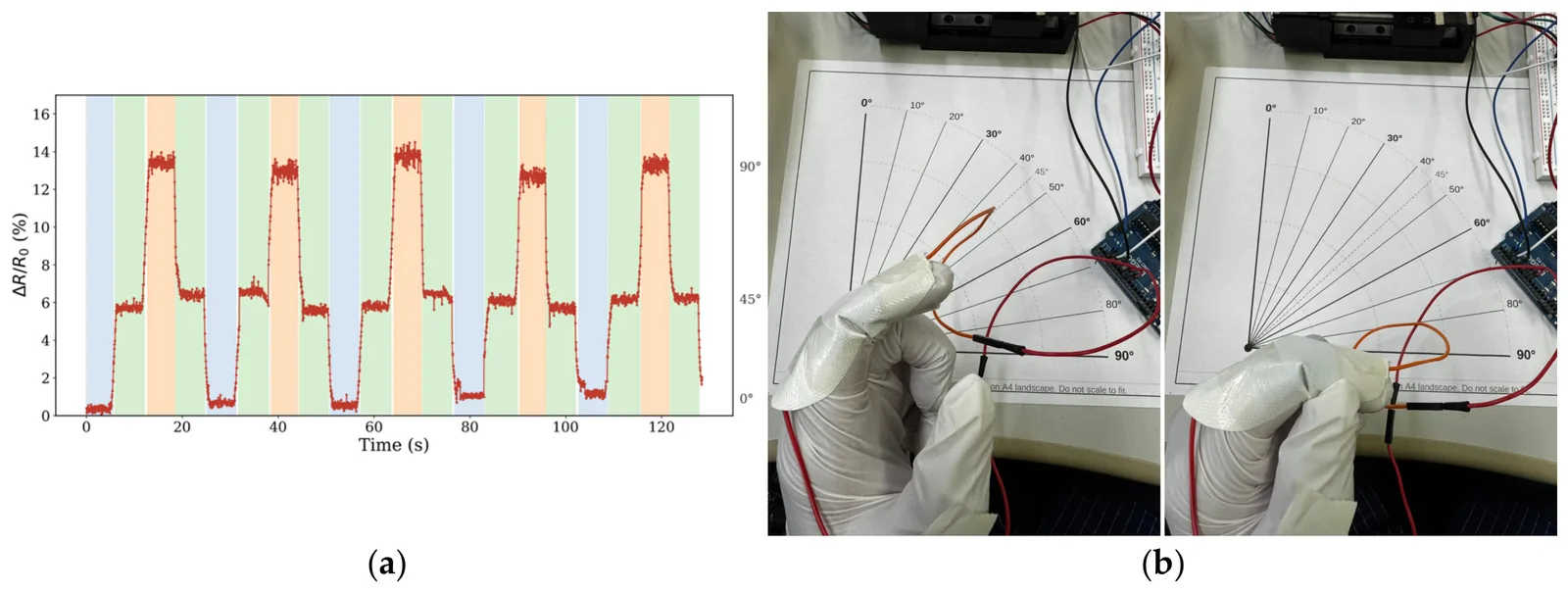
(**a**) ΔR/R_0_ of the glove-mounted DSTT sensor during manual stepping of the finger through 0°, 45°, and 90° over five repeated sequences within one continuous recording, with blue, green, and orange shaded bands indicating the nominal 0°, 45°, and 90° holding positions, respectively, and marking the twenty individual holding segments used to compute the per-angle statistics in Section 3.5; (**b**) representative photographs of the finger positioned at 45° and 90° against the printed angle template.

Figure 7 shows the response of the glove-mounted DSTT sensor when the finger was manually stepped through 0°, 45°, and 90° against a printed angle template, over repeated sequences in a continuous recording. The data show a clear three-level step response: a lower reference level corresponding to the straight (0°) finger, an intermediate plateau corresponding to 45°, and an upper plateau corresponding to 90°, with representative 45° and 90° finger positions shown in Figure 7b. The transitions between levels are sharp, and the plateaus are well separated: across the five complete 0° → 45° → 90° → 45° sequences (twenty holding segments in total), the mean response was 0.85 ± 0.37% at 0° (n = 5 segments), 6.12 ± 0.32% at 45° (n = 10), and 13.15 ± 0.38% at 90° (n = 5), where the standard deviation is computed across repeated holding segments within the same continuous experiment rather than across independent trials. The gap between adjacent angle levels exceeds 13 times the largest segment-to-segment variability, so the three levels recur consistently and are unambiguously separated across all repetitions of the sequence. The non-zero 0° response is treated as the baseline reference level of the glove measurement rather than as an enforced zero-response condition; it may reflect the initial R_0_ definition and small residual curvature of the finger/sensor assembly at the nominal 0° position.

Two aspects of this result are worth noting. First, each response level remained within a relatively narrow range across the repeated holding segments, allowing clear distinction among the manually assigned 0°, 45°, and 90° finger positions. Second, the response returned toward its baseline when the finger was straightened, consistent with the recovery behavior observed in the controlled bending tests. The angle-stepped demonstration therefore indicates that the stable baseline, repeatable response, and increasing response with bending observed on the rig are also evident under manual, wearable bending conditions.

## 4. Discussion

For the present specimens, the dynamic measurements show a clear difference between DSTT and RT under the tested protocols. DSTT retained 89.2% of its initial peak response across 1000 cycles with a peak CV of 4.3%, whereas RT retained 20.0% with a peak CV of 50.1%. This contrast can be interpreted in the context of percolation and tunneling conduction in CNT networks [25,26]. In the fabrication study, cross-sectional and surface scanning electron microscopy (SEM) showed that room-temperature drying leaves a thinner, less continuous MWCNT network, whereas DSTT combines sonication-assisted penetration with thermal consolidation to form a more interconnected coating [14]. A denser network would be expected to provide multiple parallel conduction paths, which likely contributes to the greater response stability observed for the DSTT sensor by reducing its dependence on individual CNT–CNT contacts during bending. Under static conditions, this distinction is also reflected in the roughly 65-fold sheet-resistance difference at Coating 6 [14].

The RT specimen retained 20.0% of its initial peak response after 1000 bending cycles, compared with 89.2% for the DSTT specimen. Separately measured unbent resistances increased from approximately 100 to 105 Ω for DSTT and from approximately 5.6 to 5.79 kΩ for RT. These multimeter readings characterize the before- and after-test unbent resistance states, whereas the apparent baseline shifts extracted from the cyclic recordings compare the mean cycle minima over the initial and final 20 cycles. The apparent shifts were smaller than the nominal ADC quantization increment near baseline and are therefore interpreted conservatively. The electrical measurements demonstrate different response-retention behaviors between the tested specimens but do not establish their microscopic origin. Conductive-network rearrangement remains a possible interpretation. This mechanistic interpretation is limited to the electrical evidence available here, as no post-cycling structural characterization was performed. The present measurements do not establish whether coating cracks formed during bending; references to conductive-network rearrangement are interpretations of the electrical response rather than direct observations of structural damage. The before- and after-test unbent resistance measurements provide an additional electrical comparison, but neither these measurements nor the cyclic response alone identifies the microscopic origin of the observed changes.

The DSTT waveforms also remain similar across the four tested speeds, with only a range between the highest and lowest mean peak responses equal to 11.1% of their grand mean. This behavior could reflect predominantly reversible changes in CNT–CNT spacing and tunneling resistance during bending [27]. Because the speeds were tested in a fixed ascending order, the observed 11.1% variation cannot be attributed solely to bending speed.

Table 3 compares the present results with representative wearable bending sensors [41,42,43,44,45,46,47]. These studies use different sensing structures, bending geometries, and performance definitions; sensitivity values are therefore retained in their original units, and cyclic endurance is distinguished from quantified peak-response retention. The present DSTT specimen retained 89.2% of its peak response after 1000 bending cycles, compared with 20.0% for the RT control under the same protocol. Together with the sequential-speed response, carriage-separation calibration, and hysteresis measurement, this comparison places the dynamic performance of DSTT-coated cotton within the context of existing bending-sensor approaches.

The combination of stable cyclic response, a narrow response range across the tested speed sequence, and limited loading-unloading separation is relevant to wearable sensing, where joint motion naturally occurs at different speeds. The glove demonstration further supports the practical relevance of these characteristics: the DSTT sensing element produced three clearly separated response levels during manually controlled finger motion without machine-controlled positioning.

The angle-dependent response demonstrated here also suggests a potential direction for future work. Multiple DSTT-coated sensing elements, for example one positioned on each finger of a glove, could provide simultaneous bending information from different fingers. Combined with appropriate signal-processing or classification methods, such a configuration could be investigated for gesture and motion recognition. The stable response observed for the individual DSTT sensor in the present study provides a useful basis for exploring such multi-sensor configurations.

A second direction for future work concerns the substrate. The DSTT process was developed on cotton, and its effectiveness is likely influenced by cotton-specific properties: the porous, hydrophilic fiber structure takes up the ethanol-based MWCNT ink and may facilitate sonication-assisted penetration of nanotubes into the fabric [14]. Flexible substrates of interest for other sensor formats, such as polydimethylsiloxane (PDMS), polyvinylidene fluoride (PVDF), and thermoplastic polyurethane (TPU), have substantially different surface and structural characteristics, and elastomeric substrates can also introduce viscoelastic contributions to hysteresis and rate dependence [34,35]. Therefore, extension of DSTT to such materials would require substrate-specific optimization of deposition, sonication, and thermal treatment with respect to factors such as wetting, adhesion, and thermal compatibility. The results reported here provide a dynamic-performance reference for evaluating such adapted processes.

### Limitations

The dynamic tests were designed as detailed within-specimen characterizations, while reproducibility of static electrical properties across specimens was established in the preceding study [14]. Because the speeds were tested in a fixed ascending order, the effects of bending speed and cumulative cycle history cannot be fully separated. Carriage separation was the directly controlled deformation variable, so the reported %/mm sensitivity is specific to the present test geometry rather than a conventional strain-based gauge factor. The glove angles were manually assigned using an angle template and should therefore be regarded as approximate positions. Environmental cross-sensitivity, step-response timing, long-duration static creep, and post-cycling morphology were not independently characterized in the present study. Between-specimen reproducibility of dynamic response retention, response variability, and hysteresis was not established in the present study. Hysteresis was characterized using a single complete bending and bending-back sweep; its variability across repeated sweeps and independent specimens was not established. The glove experiment demonstrates discrimination among three approximate finger positions within one continuous recording; long-duration repeated finger motion and between-sensor wearable performance were not evaluated.

## 5. Conclusions

This study demonstrates the dynamic bending-sensing capability of DSTT-fabricated MWCNT-coated cotton fabric at the Coating 6 condition previously identified as having the lowest sheet resistance [14]. The DSTT sensor retained 89.2% of its peak response after 1000 continuous bending cycles, compared with 20.0% for the RT control, and maintained regular waveforms across the 1–8 mm/s sequential speed protocol with a range between the highest and lowest mean peak responses equal to 11.1% of their grand mean. Carriage-separation testing produced closely matched bending and bending-back slopes of −0.580 and −0.581%/mm (R^2^ = 0.982–0.986) and a maximum hysteresis of 8.7% of full scale. In the glove demonstration, the DSTT sensor generated distinct response levels at manually assigned 0°, 45°, and 90° finger positions. For the tested specimens and tested protocols, DSTT showed substantially greater response retention and lower cycle-to-cycle peak variability than the RT control. The separate glove demonstration further supports the feasibility of using DSTT-coated cotton as a piezoresistive bending-sensing element. Within the present fixed-order, selected-specimen protocol, the results provide a basis for subsequent independent-device and strain-calibrated validation.

## Figures and Tables

**Table 1:** Peak response versus bending speed for the DSTT and RT sensors, with the number of cycles recorded at each speed. Mean ± standard deviation (SD) values are reported; SD denotes within-specimen cycle-to-cycle variability and does not represent variability between independently fabricated sensors. DSTT: drop-casting, sonication, and thermal treatment; RT: room-temperature-dried control.

Speed (mm/s)	n Cycles (DSTT)	DSTT Peak (%)	n Cycles (RT)	RT Peak (%)
1	4	12.27 ± 0.23	4	52.81 ± 7.26
2	8	11.95 ± 0.20	8	30.67 ± 4.29
4	16	11.40 ± 0.15	16	14.96 ± 2.56
8	32	10.98 ± 0.16	32	6.72 ± 1.13

**Table 2:** Quantitative summary of DSTT and RT dynamic performance from the multi-speed and cyclic-durability tests, together with carriage-separation sensitivity and hysteresis metrics for the DSTT sensor. Where mean ± SD values are reported, SD denotes within-specimen cycle-to-cycle variability and does not represent variability between independently fabricated sensors.

Metric	DSTT	RT
Unbent resistance before the durability test	≈100 Ω	≈5.6 kΩ
Unbent resistance after 1000 bending cycles	≈105 Ω	≈5.79 kΩ
Peak response, first 20 cycles (%)	11.54 ± 0.39	20.24 ± 6.04
Peak response, last 20 cycles (%)	10.29 ± 0.31	4.05 ± 0.24
1000-cycle retention (%)	89.2	20.0
Peak CV across 1000 cycles (%)	4.3	50.1
Apparent baseline shift across 1000 cycles (percentage points; near ADC quantization limit)	+0.22	+0.29
Peak variation across sequential speed segments, 1–8 mm/s (% of grand mean; speed and cycle history confounded, see Section 3.1)	11.1	175
Sensitivity (%/mm of carriage-separation change)	0.58 (R^2^ = 0.982–0.986)	—
Maximum hysteresis (% of full-scale response)	8.7	—

**Table 3:** Comparison of representative wearable bending sensors: sensing structures, test conditions, and electrical response.

Year	Substrate/Support	Sensing Material/Structure	Cyclic Bending Conditions	Electrical Performance (as Reported)	Rate Testing/Wearable Demonstration	Reference
2026	TPU	FDM-printed conductive TPU	700 cycles; 0–90°	Angle calibration: third-degree polynomial; R^2^ ≈ 0.90. Hysteresis: 27% FS. Peak retention: NR.	Bending speeds: 40, 65, 70, 75°/s; finger-bending application.	[41]
2021	PET	Solution-processed Cu_2_O film	1000 cycles; 1 Hz	Bending-derived gauge factors: 5.88 and 18.2 in two curvature regions. Post-fatigue resistance increase: ≈10%, distinct from peak retention. Hysteresis: NR as % FS.	Cycling frequencies: 0.15, 0.5, 1 Hz; finger and wrist demonstrations.	[42]
2023	Knitted textile	Conductive-yarn finger sensor	Bending-durability cycle count: NR	Response at 45°: 18 ± 1% without a spacer; 41 ± 2% with two spacer layers. Bending retention and hysteresis: NR.	Finger-angle testing at 0°, 45°, 90°.	[43]
2015	Paper	AgNW/LDH/WPU conductive coating	>3000 cycles; 4 Hz; bending radius 5 mm	Angular sensitivity: 0.16 rad^−1^. Peak retention and hysteresis: NR as numerical percentages.	Finger and elbow demonstrations.	[44]
2025	Parylene	Ultrathin silicon with direct Au–Au bonding	10,000 cycles at 10 mm radius; supplementary testing at 4 mm radius	Curvature sensitivity: 0.0712 per mm^−1^; R^2^ = 0.99. No noticeable output degradation reported; numerical peak retention and hysteresis: NR.	Glove-mounted finger-motion tracking.	[45]
2021	Flexible polyimide substrate	Carbon-fiber junction	10,000 cycles; 60° outward bending and release	Angle calibration: ln(1 + ΔR/R_0_), R^2^ = 0.99142. Peak retention: NR as a percentage. Hysteresis discussed without % FS.	Finger, wrist, elbow, and knee demonstrations.	[46]
2021	PET support	Lamellar graphene aerogel	10,000 cycles; 90°; 1 Hz	Angle response: nonlinear over 0–180°. Detection limit: 0.29°, distinct from sensitivity. Peak retention and hysteresis: NR as numerical percentages.	Finger-bending demonstration.	[47]
2026	Cotton fabric	DSTT-deposited MWCNT coating	1000 cycles; 4 mm/s; carriage separation ≈25–4 mm	Peak retention: 89.2%; RT control: 20.0%. Sensitivity: ≈0.58%/mm; R^2^ = 0.982–0.986. Hysteresis: 8.7% FS, single sweep at 4 mm/s.	Sequential speeds: 1, 2, 4, 8 mm/s; mean-peak variation: 11.1% of grand mean. Finger responses: 0.85 ± 0.37%, 6.12 ± 0.32%, 13.15 ± 0.38% at nominal 0°, 45°, 90°.	Present work

NR: The specified quantitative bending metric was not reported in the cited study. FS: Full-scale response. Cycle counts were obtained under different bending amplitudes, geometries, and rates. Sensitivities retain their original definitions and units. Resistance change after fatigue, peak-response retention, detection limit, and calibration linearity are distinct quantities. The present speed-segment variation includes cumulative cycle-history effects. Tensile-mode hysteresis values are excluded. FDM: fused deposition modeling; TPU: thermoplastic polyurethane; PET: polyethylene terephthalate; AgNW: silver nanowire; LDH: layered double hydroxide; WPU: waterborne polyurethane; MWCNT: multi-walled carbon nanotube.

## Data Availability

The data presented in this study are available from the corresponding author upon reasonable request.

## Abbreviations

The following abbreviations are used in this manuscript:

MWCNT	Multi-walled carbon nanotube
CNT	Carbon nanotube
DSTT	Drop-casting, sonication, and thermal treatment
RT	Room temperature (room-temperature-dried control)
PDMS	Polydimethylsiloxane
PVDF	Polyvinylidene fluoride
TPU	Thermoplastic polyurethane
ADC	Analog-to-digital converter
AgNW	Silver nanowire
CV	Coefficient of variation
FDM	Fused deposition modeling
FS	Full-scale response
JSPS	Japan Society for the Promotion of Science
LDH	Layered double hydroxide
NR	Not reported
PET	Polyethylene terephthalate
SD	Standard deviation
SEM	Scanning electron microscopy
USB	Universal serial bus
WPU	Waterborne polyurethane
